# Human tissue-specific MSCs demonstrate differential mitochondria transfer abilities that may determine their regenerative abilities

**DOI:** 10.1186/s13287-018-1012-0

**Published:** 2018-11-08

**Authors:** Swati Paliwal, Rituparna Chaudhuri, Anurag Agrawal, Sujata Mohanty

**Affiliations:** 1 Stem Cell Facility, DBT Centre of Excellence for Stem Cell Research, All India Institute of Medical Sciences, New Delhi, 110029 India; 2 Department of Bioscience and Biotechnology, Banasthali Vidyapith, Rajasthan, 304022 India; 3 Molecular Immunogenetics Laboratory and Centre of Excellence for Translational Research in Asthma & Lung Disease, CSIR-Institute of Genomics and Integrative Biology, Mall Road, Delhi, 110007 India

**Keywords:** Mitochondrial transfer, Tissue-specific MSCs, Mitochondrial bioenergetics

## Abstract

**Background:**

Recent studies have demonstrated mesenchymal stem cells (MSCs) as effective mitochondrial donors with therapeutic success in multiple experimental models of human disease. MSCs obtained from different tissue sources such as bone marrow (BM), adipose (AD), dental pulp (DP), and Wharton’s jelly (WJ) are routinely used in clinical trials with no known study of their mitochondrial donor capacity. Here, we show for the first time that MSCs derived from different tissue sources have different mitochondrial donor properties and that this is correlated with their intrinsic respiratory states.

**Methods:**

MitoTracker^®^-labeled MSCs were co-cultured with Cell Trace–labeled U87-MG cells or rat cardiomyocytes. Mitochondrial transfer abilities of MSCs were assessed by using flow cytometry analysis and fluorescence imaging. Mitochondrial reactive oxygen species (mtROS) levels were analyzed by using MitoSOX red–based staining, and mitochondrial respiration parameters were analyzed by using a Seahorse XF Analyzer.

**Results:**

AD-MSCs and BM-MSCs displayed higher mitochondrial transfer than DP-MSCs and WJ-MSCs. Counterintuitively, DP-MSCs and WJ-MSCs were more effective in suppressing mtROS levels in stressed recipient cells than AD-MSCs or BM-MSCs. Interestingly, the oxygen consumption rates and intrinsic mitochondrial respiration parameters like ATP levels, basal and maximal respiration, and mitochondrial DNA copy number in donor MSCs showed a highly significant inverse correlation with their mitochondrial donation.

**Conclusions:**

We find that there are intrinsic differences in the mitochondrial respiration, donation capacity, and therapeutic efficacy among MSCs of different tissue origin. MSCs with high mitochondrial respiration capacities are associated with lower mitochondrial transfer but more effective suppression of mtROS in stressed recipient cells. This is most compatible with a model where recipient cells optimally regulate mitochondrial transfer such that they take more mitochondria from MSCs with lower mitochondrial function. Furthermore, it appears to be advantageous to use MSCs such as DP-MSCs or WJ-MSCs with higher mitochondrial respiratory abilities that achieved better therapeutic effect with lower mitochondrial transfer in our study. This opens up a new direction in stem cell therapeutics.

**Electronic supplementary material:**

The online version of this article (10.1186/s13287-018-1012-0) contains supplementary material, which is available to authorized users.

## Background

Mesenchymal stem cells (MSCs) are now routinely used in a number of clinical trials in regenerative medicine. They are easily available from different tissue sources such as bone marrow (BM), adipose (AD), dental pulp (DP), and Wharton’s jelly (WJ) [1]. Results from a number of clinical trials have shown that MSCs hold huge promise for amelioration of several diseases for which no effective cure is available to date. However, the choice of parameters for determining the optimum tissue source and donor for efficient successful therapeutic outcome remains understudied.

MSCs function through several mechanisms such as inter-cellular mitochondrial transfer, paracrine effect, and direct differentiation to regenerate damaged tissue [2, 3]. MSCs have been shown to exert a beneficial effect through mitochondrial transfer in many diseases such as stroke, asthma, and cardiac diseases [4, 5]. MSCs transfer their healthy mitochondria to repair and rescue dysfunctional mitochondria in damaged cells. Spees et al. demonstrated for the first time that mitochondrial transfer from healthy MSCs could rescue cells with non-functional mitochondria and restore their aerobic respiration [6]. Recent advances have demonstrated that stressed cells release certain environmental cues that trigger mitochondrial transfer from MSCs in co-culture [7–10]. Damaged mitochondria and mitochondrial products such as damage-associated molecular patterns (DAMPs) are released as stress signals during cellular injury. These factors, along with elevated levels of reactive oxygen species levels, signal the MSCs to enhance their bioenergetics and initiate mitochondria donation to injured recipient cells [10, 11]. A majority of studies have shown that mitochondria transfer from MSCs to damaged cells occurs through tunneling nanotubes that form the inter-cellular connections between the donor and recipient cell [12–15]. Nonetheless, several other modes of mitochondrial transfer such as formation of cell junctions, microvesicles, cell fusion, and transfer of isolated mitochondria can also mediate mitochondrial transfer to repair and regenerate damaged recipient cells [3, 4, 16–19]. Mitochondrial transfer from BM-MSCs has been shown to protect mice from lipopolysaccharide (LPS)-induced acute lung injury [16]. MSCs have also shown the potential to reprogram adult cardiomyocytes to a progenitor-like state through cell fusion and cell-to-cell connection that facilitate mitochondrial transfer in ischemic cardiomyoblasts [17, 20, 21]. An upsurge of recent studies has shown successful therapeutic effectiveness of mitochondrial transfer in regeneration of various recipient cells such as damaged corneal epithelium cells, renal tubular cells, brain cortical cells, neurons, alveolar cells, and cardiomyocytes [4, 5, 9]. MSCs isolated from BM-MSCs, AD-MSCs, WJ-MSCs, and DP-MSCs have all been used for transplantation into patients in many clinical trials [4, 22]. Studies have shown that these MSCs from these different tissue origins demonstrate the ability to transfer mitochondria to rescue damaged cells and restore respiratory potential in recipients [17, 23]. However, to date, no study has compared the mitochondrial transfer efficiencies of tissue-specific MSCs to injured cells with dysfunctional mitochondria. In this study, we investigate the differential mitochondria transfer abilities of MSCs derived from BM-MSC, AD-MSC, DP-MSC, and WJ-MSC sources to stressed U87-MG cells or rat cardiomyocytes, treated with antimycin. Here, we have also compared the rescue potential of tissue-specific MSCs indicated by their abilities to reduce mitochondrial reactive oxygen species (mtROS) levels in recipient cells under oxidative stress. Mitochondrial parameters such as membrane potential, basal mtROS, mitochondrial biomass, mitochondrial DNA (mtDNA) copy number, and ATP levels were also compared between various tissue-specific MSCs to understand the contributing factor for the differences in mitochondrial transfer abilities. This study suggests that mitochondrial parameters of tissue-specific MSCs are critical variables in determining the optimum tissue source of MSCs for regenerative therapy purposes.

## Methods

### Revival and expansion of cryopreserved human MSCs

Cryopreserved human BM-MSCs, AD-MSCs, DP-MSCs, and WJ-MSCs (n = 4 or 6 each) were selected. These samples were obtained after receiving prior informed consent from the donors. MSCs at passage 3 were revived in low-glucose culture medium containing Dulbecco’s modified Eagle’s medium (LG-DMEM) (Gibco, Gaithersburg, MD, USA) containing 10% fetal bovine serum (HyClone, Logan, UT, USA) supplemented with 1% Glutamax and Penstrep (Invitrogen, Waltham, MA, USA), incubated at 37 °C with 5% CO_2_. *In vitro* culture expansion and characterization of MSCs and viability test were carried out in accordance with previously described lab protocol [24]. Cells at 75–80% confluency were used for further experiments. After revival, the cell sample was diluted in a 1:1 dilution using 0.4% Trypan blue solution; 10 μL of this dilution was loaded in a hemocytometer, and viability was confirmed immediately under microscope.

### Characterization of the cultured cells

#### Surface marker analysis through flow cytometry

Single-cell suspensions of MSCs from all of the sources were prepared in media after detaching the cells from the flask using TrypLE Express. The cells at a concentration of 0.5–1 × 10^6^ per mL were stained with labeled antibodies for surface markers CD105, CD29, CD73, CD90, HLAI and HLAII, and hematopoetic marker CD34/45. These were incubated at room temperature for 1 h. Corresponding isotypes: IgG1 coupled with PE, PECy5, APC, and FITC were used as controls. Characterization of the cultured cells was performed at the third passage. The cells were acquired on a BD LSR II flow cytometer and analyzed by using FACS DIVA software as per Dominici et al., 2006 [25]. Table 1 shows surface marker characterization of representative tissue-specific MSCs.

**Table 1:** Surface marker characterization of tissue-specific mesenchymal stem cells (expressed in percentages)

S. no.	Cell type	CD105	CD73	CD90	CD29	HLA-I	HLA-II	CD34/45
1	BM-MSCs	86.9	80	91.1	72.2	85.7	0	2.5
2	BM-MSCs	81.8	81	90	76	83.8	0.7	0
3	BM-MSCs	84.6	82.9	91.8	75.9	81	1.6	0
4	AD-MSCs	86.7	74.9	71.2	44.5	83.6	0	0
5	AD-MSCs	75.2	69.8	84.5	70.9	79.2	1.1	2.2
6	AD-MSCs	76.8	73.1	81.1	68.8	75.3	0.5	0
7	DP-MSCs	80.6	87.5	78.1	79.6	82.5	1.1	2.2
8	DP-MSCs	86	85.2	83	82.1	80.7	0	0
9	DP-MSCs	84.3	83.3	60.9	84.4	92.2	3.3	0
10	WJ-MSCs	71.6	79.4	85.2	83.3	77.2	0	0.1
11	WJ-MSCs	82	96.9	98.1	92.4	73.6	0.1	0
12	WJ-MSCs	87	96.8	98.2	92.1	73	0	0

Abbreviations: *AD-MSC* adipose-mesenchymal stem cell, *BM-MSC* bone marrow-mesenchymal stem cell, *DP-MSC* dental pulp-mesenchymal stem cell, *WJ-MSC* Wharton’s jelly-mesenchymal stem cell

#### Trilineage differentiation

MSCs were induced for trilineage differentiation (osteogenesis, adipogenesis, and chondrogenesis) and cells showed successful differentiation to these three lineages as indicated by specific staining for every lineage [26].

### Co-cultures of MSCs with stressed cells

Tissue-specific MSCs (BM-MSCs, AD-MSCs, DP-MSCs, and WJ-MSCs) were labeled with 100 nM MitoTracker^®^ Green FM (Thermo Fisher Scientific, Waltham, MA, USA) in accordance with the protocol of the manufacturer. U87-MG and rat cardiomyocytes were labeled with Cell Trace Violet™ (Thermo Fisher Scientific) at a 5-μM concentration in accordance with the protocol of the manufacturer. Two media washes were given to remove any unbound reagent. Tissue-specific MSCs were trypsinized and seeded onto wells containing antimycin-treated U87-MG or rat cardiomyocytes at a 1:1 ratio containing equal amounts of respective media. The percentage transfer of mitochondria from MSCs to stressed recipients was calculated after 24 h of co-culture. The cells were assessed for various parameters by confocal imaging or flow cytometry analysis.

### Oxidative stress induction

Antimycin A (Sigma-Aldrich, St. Louis, MO, USA) at 100 nM was added to culture media of U87-MG or cardiac cells (50,000 cells per well in a 12-well culture plate) and was further incubated for 16–18 h to induce oxidative stress prior to adding MSCs in the co-culture system.

### Live cell microscopy

Live cell imaging was carried out by confocal microscope (Leica TCS SP5; Leica, Wetzlar, Germany). Live cell microscopy was carried out in cells seeded in two- or four-chamber glass slides (Nunc™ Lab-Tek™ II Chamber Slides, Thermo Scientific Fisher, USA) under optimal culture conditions, with 37 °C temperature and 5% CO_2_. Cells were imaged 24 h after co-culture. All images showing mitochondria transfer were captured with either 100× objective (DMI6000) or 63× lambda blue objective (SP5).

### Mitochondria parameters assessment by flow cytometry

MitoTracker^®^ Green FM (excitation/emission: 419/560 nm) was used for mitochondrial biomass quantification in MSCs. Tetramethylrhodamine ethyl ester perchlorate (TMRE) (excitation/emission: 550/575 nm) was used at a concentration of 100 nM and incubated for 20 min at 37 °C for assessment of membrane potential of MSCs. mtROS was measured by using MitoSOX red (Thermo Fisher Scientific) at a concentration of 4 μM for 20 min and incubated at 37 °C with 5% CO_2_. Quantification of mtROS was carried out by using a BD LSR II flow cytometer (Becton Dickinson, Franklin Lakes, NJ, USA) with at least 10,000 events for each sample and analyzed with Becton Dickinson FACS Diva (version 6.1.2). Readings (in duplicates) for mean fluorescence intensity (MFI) in the PE region were recorded in arbitrary units (AU).

### Genomic DNA isolation

Genomic DNA was isolated from tissue-specific MSCs for mtDNA copy number analysis by using a GeneJET Genomic DNA extraction kit (Thermo Fisher Scientific) in accordance with the instructions of the manufacturer; 5 × 10^6^ MSCs were processed for the same.

### Measurement of mitochondrial respiration

MSCs from all four tissue origins used in this study were seeded on 24-well XF-24 plates (Seahorse Biosciences, Billerica, MA, USA) at a population density of about 60,000 per well in DMEM complete media. Oxygen consumption rate (OCR) was measured in all three groups by using the XFe24 Extracellular Flux Analyzer (Seahorse Biosciences). OCR measurements were acquired in the presence of 10 mM glucose before (basal OCR) and after mitochondrial respiration inhibitors were injected in the system. The following inhibitors—9 μM oligomycin, 0.3 μM carbonyl cyanide *p*-trifluoromethoxyphenylhydrazone, 11 μM antimycin, and 11 μM rotenone—were used to determine basal respiration, ATP production, maximal respiration, and spare respiratory capacity. These values were normalized for total protein content per well.

ATP levels were analyzed by using an ATP Assay Kit in accordance with the instructions of the manufacturer (Sigma-Aldrich) in the desired samples (5 × 10^6^ cells were used per sample), and colorimetric detection was carried out by using a spectrophotometer (BioTek, Winooski, VT, USA) at 570 nm.

### MtDNA copy number assessment

Real-time polymerase chain reaction (PCR) was performed by using an Eppendorf Thermocycler machine. Genomic DNA at a concentration of 50 ng was used for PCR reaction using Kappa SYBR Master mix (5 μl) and primers at a concentration of 0.3 μM in a 10 μl total reaction mixture. Primers used for mtDNA are forward primer: 5′-CGAAAGGACAAGAGAAATAAGG-3′ and reverse primer: 5′-CTGTAAAGTTTTAAGTTTTATGCG-3′ and nuclear housekeeping gene beta-actin primers 5′-TCACCCACACTGTGCCCATCTAGGA-3′ and 5′-CAGCGGAACCGCTCAT TGCCAATGG-3′. For PCR conditions: denaturation at 95 °C for 5 min and 40 cycles of denaturation of 15 s at 95 °C, annealing 20 s at 51.2 °C (mtDNA)/57.3 °C (nuclear beta-actin gene) and extension 15 s at 68 °C. The analysis of mtDNA copy number of the mtDNA and nuclear DNA was calculated by using threshold cycle (Ct) number. The reactions for each of the tissue-specific MSCs were carried out in duplicate by obtaining the average value of expression of mitochondrial and nuclear genes. Delta Ct (∆C) was calculated by using the following equation: Ct (mitochondrial gene) − Ct (nuclear gene). Relative mtDNA copy number was calculated by the 2^∆ct^ method.

### Statistical analysis

Data are presented as mean ± standard error of mean. Statistical analyses were performed by using either one-way or two-way analysis of variance (ANOVA), Tukey’s post hoc analysis, or unpaired Student’s *t* test. Statistical significance was set at a *P* value of less than 0.05 to study differences between donor MSCs. Graphs were plotted by using GraphPad PRISM 7.0 or R 2.13.0 (GraphPad Software, La Jolla, CA, USA) [27].

## Results

### Mitochondrial transfer from MSCs to U87-MG and rat cardiac cells

**Fig. 1:**
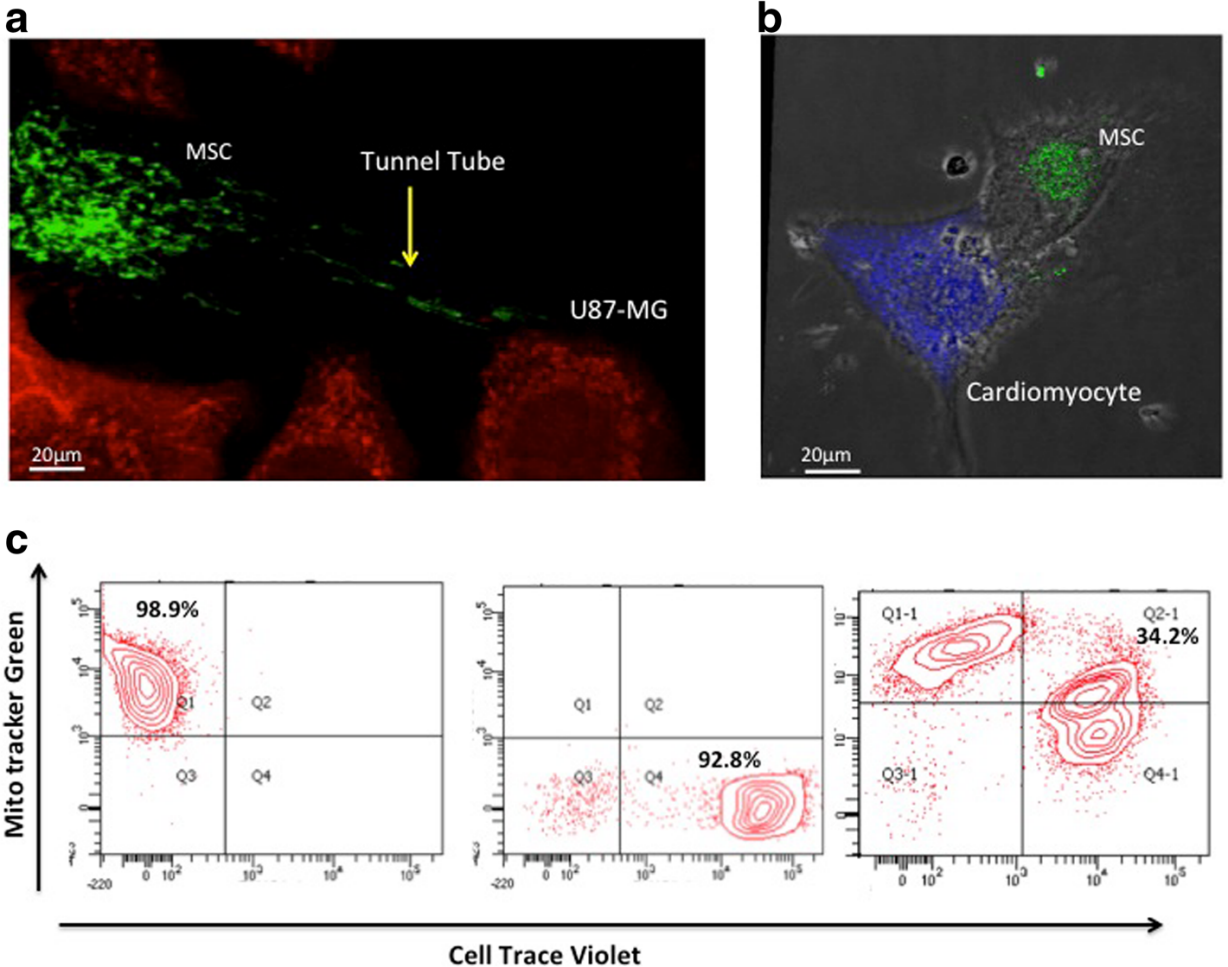
Human mesenchymal stem cells (MSCs) can transfer mitochondria to U87-MG cells and rat cardiomyocytes. (**a**) Representative confocal images of mitochondrial transfer from bone marrow–MSCs (labeled with MitoTracker^®^ Green) to U87-MG cells and (**b**) rat cardiomyocytes (labeled with Cell Trace Violet shown in red and violet, respectively). Scale bar = 20 μm. (**c**) A representative flow cytometric data plot shows the percentage of recipient U87-MG cells that take up mitochondria from BM-MSCs. The first plot shows cells stained with only mitotracker labeled MSC cells in Q1 quadrant, second plot shows only cell trace labeled recipient U87-MG cells in Q3 quadrant and third plot shows double positive U87-MG cells in Q2 quadrant

Revival and characterization of cryopreserved tissue-specific MSCs were performed. All MSCs used for the experiments were characterized for specific markers by using flow cytometry (representative data shown in Table 1). The patient-specific details of each tissue source are provided in supplementary data (Additional file 1: Table S1). Successful mitochondria transfer from tissue-specific MSCs to U87-MG cells and cardiomyocytes was observed when MSCs were co-cultured with the recipient cells. A representative image showing transfer of MitoTracker^®^ Green–labeled mitochondria from BM-MSCs to U87-MG cells (Fig. 1a) and cardiomyocytes (Fig. 1b) demonstrates transfer of mitochondria from MSCs to injured recipient cells in co-cultures. A representative flow cytometric data plot shows the percentage of recipient U87-MG cells that uptake mitochondria from BM-MSCs (Fig. 1c). We observed efficient labeling of Mitochondria Tracker Green in MSC cultures and Cell Trace Violet in U87-MG cells, and the transfer of mitochondria was confirmed by the presence of dual-positive cells in co-cultures composed of stained MSCs and U87-MG cells.

### Differential mitochondrial transfer from tissue-specific MSCs to recipient cells

**Fig. 2:**
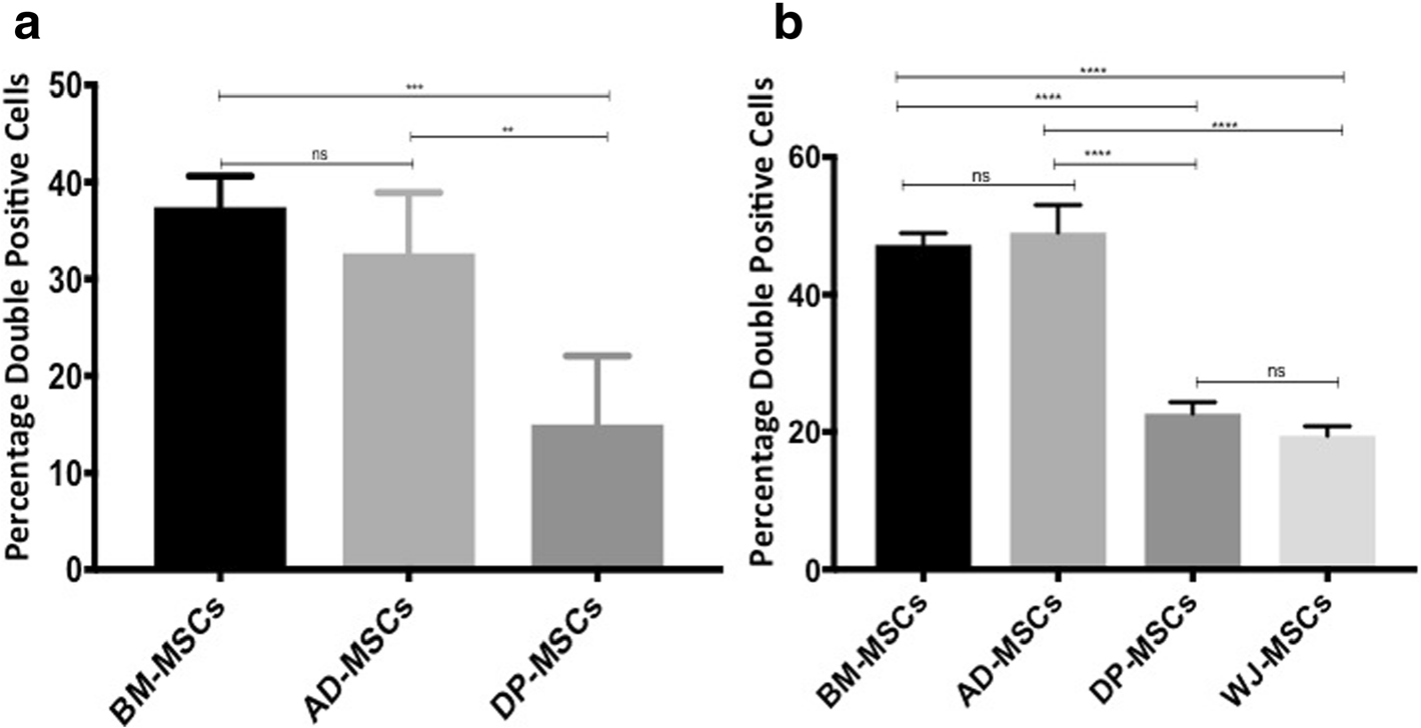
Tissue-specific mesenchymal stem cells (MSCs) demonstrate differential mitochondrial transfer under oxidative stress. (**a**) Differential mitochondrial transfer from bone marrow (BM)-MSCs, adipose (AD)-MSCs, and dental pulp (DP)-MSCs is shown in U87-MG determined by flow cytometry. Data are expressed as percentage double-positive cells, mean ± standard error of the mean (SEM) (n = 4), *P <0.05, **P <0.01, ***P <0.001 by one-way analysis of variance (ANOVA) followed by post hoc Tukey analysis. (**b**) Differential mitochondrial transfer from BM-MSCs, AD-MSCs, DP-MSCs, and Wharton’s jelly (WJ)-MSCs is shown in U87-MG determined by flow cytometry. Data are expressed as percentage double-positive cells, mean ± SEM (n = 4), *P <0.05, **P <0.01, ***P <0.001 by one-way ANOVA followed by post hoc Tukey analysis. Abbreviation: *ns* non-significant

The percentage of cells taking up mitochondria from tissue-specific MSCs was assessed by analyzing the percentage of double-positive cells in co-cultures by using flow cytometry plots (Fig. 1), and bar graphs were plotted to demonstrate differential mitochondria transfer (Fig. 2). Mitochondrial transfer from BM-MSCs and AD-MSCs was found to be significantly higher than that of DP-MSCs in U87-MG co-cultures (Fig. 2a). No significant difference was observed between mitochondrial uptake from BM-MSCs and AD-MSCs (Fig. 2a). Accordingly, BM-MSCs and AD-MSCs also transferred significantly higher percentages of mitochondria compared with DP-MSCs and WJ-MSCs in rat cardiomyocyte co-cultures (Fig. 2b). However, no difference was observed in mitochondrial donation, either between BM-MSCs and AD-MSCs or between DP-MSCs and WJ-MSCs (Fig. 2b).

### Tissue-specific MSCs differ in their abilities to reduce mtROS levels in rat cardiomyocytes

**Fig. 3:**
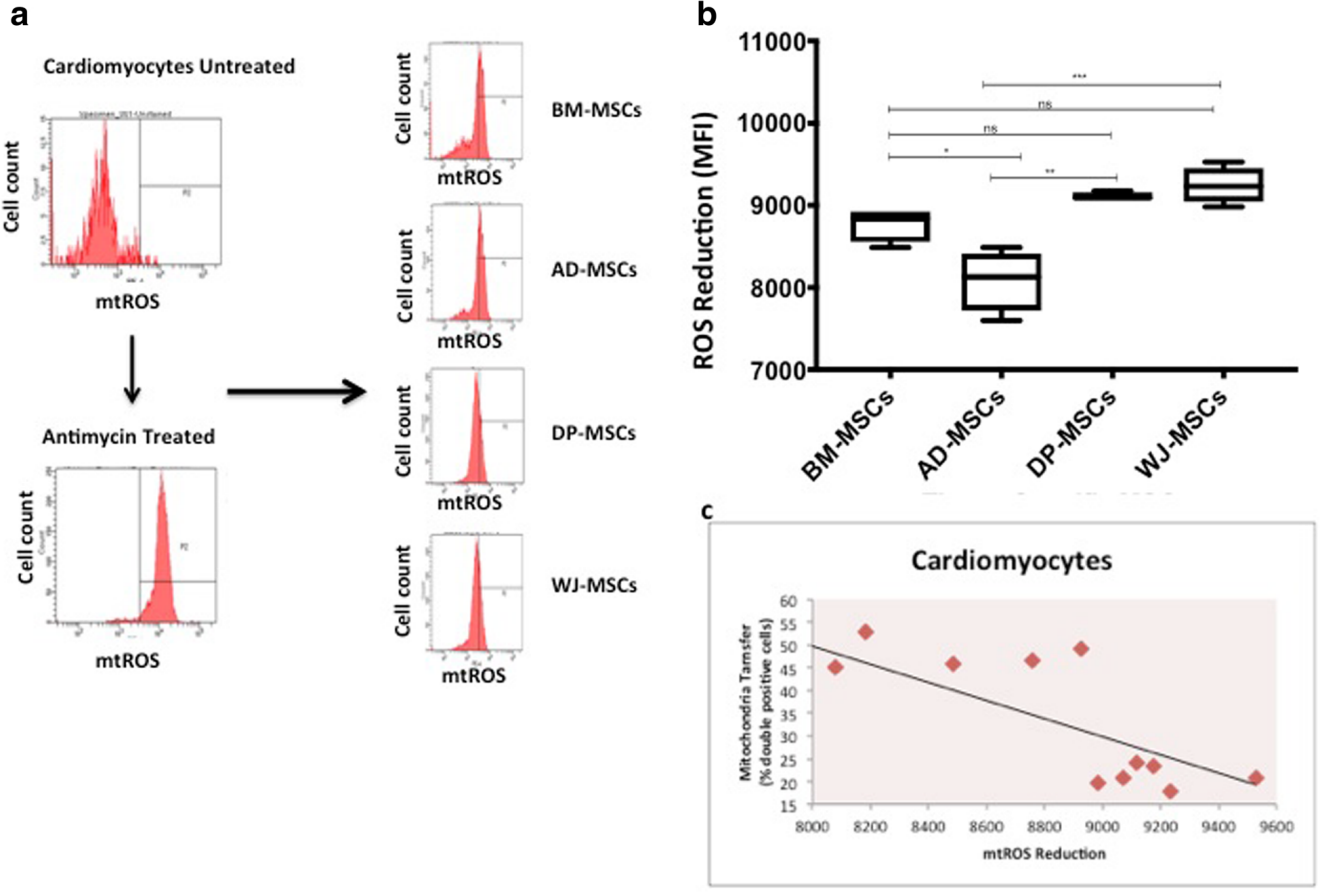
Differential reduction in reactive oxygen species (ROS) levels by tissue-specific mesenchymal stem cells (MSCs). (**a**) Representative flow cytometry data depicting mean fluorescence intensity (MFI) of ROS values indicated in arbitrary units in MitoSOX red–stained untreated and antimycin-treated cardiomyocytes, co-cultured with tissue-specific MSCs (left panel). (**b**) Bar graphs depicting reduction in ROS for bone marrow (BM)-MSCs, adipose (AD)-MSCs, dental pulp (DP)-MSCs, and Wharton’s jelly (WJ)-MSCs when co-cultured with stressed cardiomyocytes (right panel) determined by flow cytometry. Data are expressed as mean ± standard error of the mean (SEM) (n = 4), *P <0.05, **P <0.01, ***P <0.001 by one-way analysis of variance (ANOVA) followed by post hoc Tukey analysis. (**c**) Correlation between reduction in mitochondrial ROS (mtROS) reduction and mitochondrial transfer percentages (percentage double-positive cells) in cardiomyocyte co-cultures determined by Pearson’s correlation (*P* <0.05). Abbreviation: *ns* non-significant

MSCs are known to mitigate oxidative damage by lowering mtROS levels and activating anti-oxidant mechanisms for promoting healing and regeneration of damaged cells [4, 28, 29]. We observed that tissue-specific MSCs differed in their abilities to reduce elevated levels of mtROS in cardiomyocytes (Fig. 3). WJ-MSCs and DP-MSCs reduced mtROS more effectively as compared with both BM-MSCs and AD-MSCs in cardiomyocyte co-cultures (Fig. 3b). However, mtROS reduction capabilities of DP-MSCs and WJ-MSCs were found to be comparable, as were those of BM-MSCs and AD-MSCs (Fig. 3b). We further analyzed the data statistically by using the Pearson correlation test to study the correlation between percentage of cells that take up mitochondria and reduction in mtROS. A strong negative correlation between mitochondrial transfer and mtROS reduction was observed between tissue-specific MSCs in MSC-cardiomyocyte co-cultures (Pearson correlation coefficient, r square: −0.78) (*P* <0.002) (Fig. 3c). This indicates that although DP-MSCs and WJ-MSCs transferred mitochondria to a smaller number of recipient cardiomyocytes, they brought about higher reduction in overall mtROS levels as compared with BM-MSCs and AD-MSCs in oxidatively stressed cells (*P* <0.05).

### ATP bioenergetics and mitochondrial respiratory parameters may contribute to differential mitochondrial transfer

**Fig. 4:**
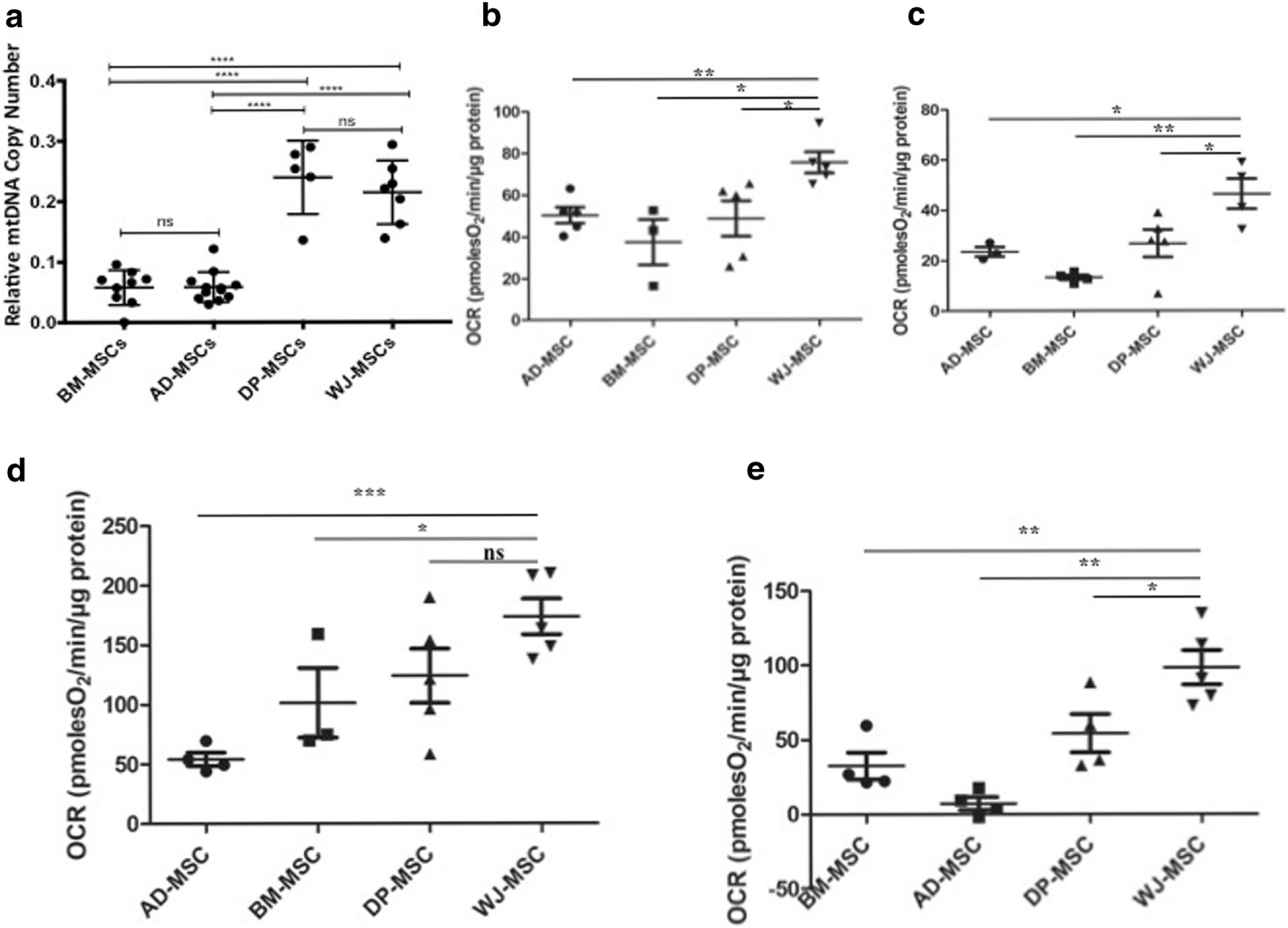
Mitochondrial parameters related to bioenergetics of tissue-specific mesenchymal stem cells (MSCs). (**a**) Mitochondrial DNA copy number (n = 6) and mitochondrial respiration parameters (**b**) basal respiration, (**c**) ATP production, (**d**) maximal respiratory capacity, and (**e**) spare respiratory capacity were measured by using a Seahorse Bioanalyzer. Data are expressed as mean ± standard error of the mean (n = 3). *P <0.05, **P <0.01, ***P <0.001 determined by unpaired Student’s *t* test. Abbreviations: *AD-MSC* adipose-mesenchymal stem cell, *BM-MSC* bone marrow-mesenchymal stem cell, *DP-MSC* dental pulp-mesenchymal stem cell, *ns* non-significant, *OCR* oxygen consumption rate, *WJ-MSC* Wharton’s jelly-mesenchymal stem cell

We studied mitochondrial copy number to investigate the gene expression of mtDNA with respect to nuclear gene expression to understand the level of mitochondrial biogenesis in various tissue-specific MSCs. We observed that DP-MSCs and WJ-MSCs have significantly higher relative mtDNA copy number compared with BM-MSCs and AD-MSCs (Fig. 4a). To test whether the intrinsic mitochondrial respiration also varies among tissue-specific MSCs, we measured all relevant mitochondrial respiration parameters like ATP production, maximal and spare respiratory capacity, and basal respiration. Interestingly, all parameters were observed to be lower in BM-MSCs and AD-MSCs compared with that in DP-MSCs and WJ-MSCs (Fig. 4b–e). We also analyzed mitochondrial biomass, basal mtROS levels, and mitochondrial membrane potential in tissue-specific MSCs but did not find any significant difference in these parameters (Additional file 2: Figure S1 a–c). Corroborating the data shown in Fig. 4, we further found a significant difference in the ATP levels between tissue-specific MSCs using an ELISA (enzyme-linked immunosorbent assay)-based ATP assay (Additional file 2: Figure S1d). DP-MSCs and WJ-MSCs displayed higher ATP levels than BM-MSCs and AD-MSCs (Additional file 2: Figure S1d). Our data confirmed that lower mitochondrial donation took place when the donor MSCs had robust mitochondrial activity in terms of their respiratory capacities (DP-MSCs and WJ-MSCs). This was in contrast to higher mitochondrial uptake from BM-MSCs and AD-MSCs that demonstrated lower ATP levels and respiratory capacities.

## Discussion

Mitochondrial transfer through MSCs has shown tremendous promise in repair and regeneration of various damaged tissues and in disease models [4, 9, 30]. It has been observed that mitochondrial transfer from MSCs occurs through various modes of transfer such as tunnel tube formation, microvesicles, gap junctions, and cell fusion [4, 5, 31]. The regenerative potential of mitochondria transfer has been observed in cases of asthma, stroke, and myocardial infarction [4, 5, 31]. Although most such studies have used BM-MSCs, other tissue sources like AD-MSCs, DP-MSCs, and WJ-MSCs can also exhibit great regenerative capacities [9, 32]. But regenerative capacity in terms of mitochondrial transfer potential of MSCs obtained from these different tissue sources has not been well explored.

In this study, we compared mitochondria donation capacities of tissue-specific MSCs obtained from different tissue sources and investigated their regenerative potentials. Studies show that MSCs reduce mtROS levels during the repair mechanisms [4, 5, 33]. Thus, we used this parameter as an index of the MSCs’ rescue ability. Our study is the first of its kind to explore the relationship between mitochondrial donation capacity and the differential rescue potential of MSCs from different sources.

A potential confounding factor in our study could be that there are age differences among the donor tissue-specific groups; however, these differences reflect the reality of the type of sources and hence could not be eliminated. In addition, we had mixed donors comprising both genders. Since WJ is obtained from the umbilical cord, all donors were female, whereas all of the donors in BM were male. Despite having mixed donors (both male and female) in AD and DP (as shown in Additional file 1: Table S1), we consistently found that mitochondrial donation was lower and intrinsic respiratory capacity was higher in DP and WJ and vice versa. This indicates that although the gender of the donor can cause large individual differences, our findings with regard to mitochondrial donation capacity and intrinsic respiratory capacity are significant enough to have overridden such individual variations. Unfortunately, it is difficult to obtain MSC donors, so we limited the sample number to between four and six per experiment. However, we believe that our findings will be applicable to a variety of donors with tissue source being the major determining factor.

We have previously shown that differential reduction of reactive oxygen species is observed when human tissue-specific MSCs under oxidative stress are co-cultured with human cardiomyocytes [28]. From our current data, it appears that differences in recipient cell type at the species level do not significantly affect mitochondrial transfer properties of donor MSCs or mtROS reduction potential as similar phenomena are observed from human MSCs to both human cell line and rat cardiomyocyte co-cultures, suggesting that it is a highly conserved and regulated process. Uptake of donor mitochondria by cardiomyocytes under oxidative stress is justified by their need to accept foreign mitochondria to meet the high energy demands of various reparative mechanisms and restore their bioenergetics profile. Relative mtDNA copy number was used to assess the expression levels of genes involved in mitochondrial bioenergetics. Higher mitochondrial respiratory capacities and elevated levels of relative mtDNA level were found in DP-MSCs and WJ-MSCs as compared with BM-MSCs and AD-MSCs. This is in accordance with studies in which an increase in mtDNA copy number suggests the need of gene expression to keep up with high bioenergetics needs in oxidative phosphorylation assembly [34].

Overall, our data suggest that bioenergetics of donor mitochondria may be a critical determining factor regulating differential mitochondrial transfer from tissue-specific donor MSCs. Our data are novel and show that the MSC source significantly determines its mitochondrial donor property and correlates with tissue rescue and intrinsic respiratory states. Thus, mitochondrial parameters and mitochondrial transfer abilities are important parameters that should be considered while choosing an optimum tissue source for regeneration of damaged cells in clinical application.

## Conclusions

Our study showed that tissue-specific MSCs demonstrate differential mitochondria transfer along with differential mtROS reduction in cells under oxidative stress. An inverse relation between mitochondrial transfer and mtROS reduction abilities of tissue-specific MSCs was observed. In addition, higher mitochondrial respiratory capacities were observed in MSCs that donated a lower percentage of mitochondria. It was observed that donor DP-MSCs and WJ-MSCs exhibit higher mitochondrial bioenergetics and robust respiratory capacities with lower mitochondrial transfer compared with BM-MSCs and AD-MSCs. This suggested that MSCs that exhibit higher mitochondrial bioenergetics and robust respiratory capacities are able to achieve higher rescue potential with lower mitochondrial transfer. Thus, this study provides important insight in determining the parameters that should be considered when choosing the optimum source of MSCs for clinical purposes in regenerative medicine.

## Additional files

Additional file 1: **Table S1.** Age and Gender Details of Tissue-Specific MCSs used in this study. (DOCX 14 kb)

Additional file 2: **Figure S1.** Mitochondrial Parameters Assessment (a) mitochondrial biomass determined by Mitotracker green calculated using flow cytometry, (n = 6). (b) Mitochondrial membrane potential assessed by Tetramethylrhodamine, ethyl ester (TMRE) using flow cytometry, (n = 6). (c) ATP assessment confirmation using ATP assay kit (Sigma Aldrich, USA), Data is expressed as mean ± SEM (n=3). *P < 0.05, **P < 0.01, ***P < 0.001. (JPG 48 kb)

## Abbreviations

AD: Adipose
BM: Bone marrow
CD: Cluster of differentiation
Ct: Threshold cycle
DAMP: Damage-associated molecular pattern
DMEM: Dulbecco’s modified Eagle’s medium
DP: Dental pulp
HLA: Human leukocyte antigen
MSC: Mesenchymal stem cell
mtDNA: Mitochondrial DNA
mtROS: Mitochondrial reactive oxygen species
OCR: Oxygen consumption rate
PCR: Polymerase chain reaction
ROS: Reactive oxygen species
WJ: Wharton’s jelly
