# Correction to: Human tissue-specific MSCs demonstrate differential mitochondria transfer abilities that may determine their regenerative abilities

**DOI:** 10.1186/s13287-019-1343-5

**Published:** 2019-07-26

**Authors:** Swati Paliwal, Rituparna Chaudhuri, Anurag Agrawal, Sujata Mohanty

**Affiliations:** 1 Stem Cell Facility, DBT Centre of Excellence for Stem Cell Research, All India Institute of Medical Sciences, New Delhi, 110029 India; 2 Department of Bioscience and Biotechnology, Banasthali Vidyapith, Rajasthan, 304022 India; 3 Molecular Immunogenetics Laboratory and Centre of Excellence for Translational Research in Asthma & Lung Disease, CSIR-Institute of Genomics and Integrative Biology, Mall Road, Delhi, 110007 India

**Correction to: Stem Cell Res Ther 2018;9:298.**

**https://doi.org/10.1186/s13287-018-1012-0**

The original article [1] contains errors in Fig. 1. The authors noticed a potentially misleading aspect of the original article Fig. 1 where representative flow cytometry data for different panels were from different data sets and thus the gates were not in the same line. This may cause confusion to the readers who attempt to compare panels and, thus the amended Fig. 1 shown ahead represents data from a single data set that is suitable for between panel comparisons.

**Fig. 1:**
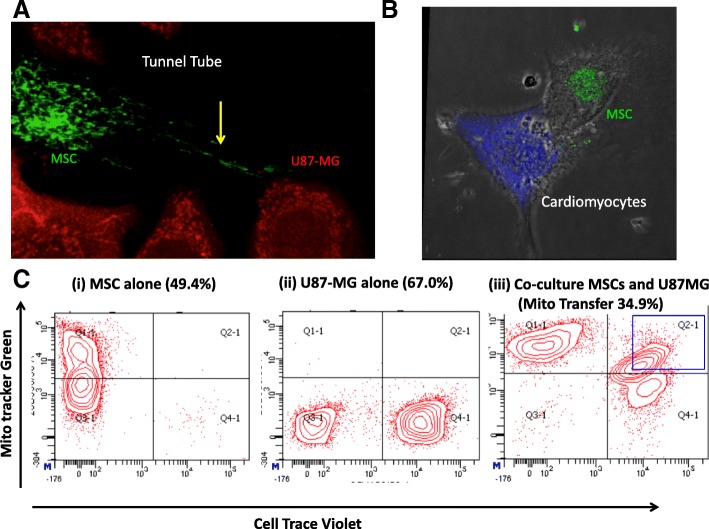
Human mesenchymal stem cells (MSCs) can transfer mitochondria to U87-MG cells and rat cardiomyocytes. **a** Representative confocal images of mitochondrial transfer from bone marrow–MSCs (labeled with MitoTracker® Green) to U87-MG cells and (**b**) rat cardiomyocytes (labeled with Cell Trace Violet shown in red and violet, respectively). Scale bar = 20 μm. **c** A representative flow cytometric data plot shows the percentage of recipient U87-MG cells that take up mitochondria from BM-MSCs. The first plot shows cells stained with only mitotracker labeled MSC cells in Q1 quadrant, second plot shows only cell trace labeled recipient U87-MG cells in Q3 quadrant and third plot shows double positive U87-MG cells in Q2 quadrant
