# Supplementary material for: Human tissue-specific MSCs demonstrate differential mitochondria transfer abilities that may determine their regenerative abilities
Source: Stem Cell Res Ther. 2018 Nov 8;9:298. doi: 10.1186/s13287-018-1012-0 (PMC6225697; doi:10.1186/s13287-018-1012-0)
Supplement: Supplementary file 1 — Table S1. Age and Gender Details of Tissue-Specific MCSs used in this study. (DOCX 14 kb) [file 13287_2018_1012_MOESM1_ESM.docx]

**Table S1: Age and Gender Details of Tissue-Specific MCSs used in this study**

| **S.No** | **Tissue source** | **Age/Gender** |
| --- | --- | --- |
| 1 | BM-MSC | 41/M |
| 2 | BM-MSC | 45/M |
| 3 | BM-MSC | 52/M |
| 4 | BM-MSC | 47/M |
|  |  |  |
| 5 | DP-MSC | 14/M |
| 6 | DP-MSC | 10/M |
| 7 | DP-MSC | 14/M |
| 8 | DP-MSC | 17/F |
| 9 | DP-MSC | 14/F |
|  |  |  |
| 10 | WJ-MSC | 24/F |
| 11 | WJ-MSC | 28/F |
| 12 | WJ-MSC | 30/F |
| 13 | WJ-MSC | 35/F |
| 14 | WJ-MSC | 32/F |
| 15 | WJ-MSC | 29/F |
|  |  |  |
| 16 | AD-MSC | 27/F |
| 17 | AD-MSC | 29/F |
| 18 | AD-MSC | 21/M |
| 19 | AD-MSC | 27/M |
