# Supplementary figures and images for: Human tissue-specific MSCs demonstrate differential mitochondria transfer abilities that may determine their regenerative abilities
Source: Stem Cell Res Ther. 2018 Nov 8;9:298. doi: 10.1186/s13287-018-1012-0 (PMC6225697; doi:10.1186/s13287-018-1012-0)

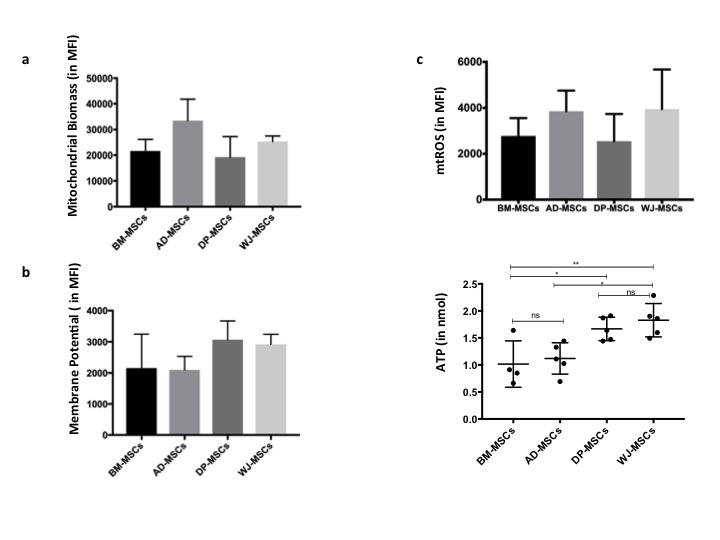

Supplement: Supplementary file 2 — Figure S1. Mitochondrial Parameters Assessment (a) mitochondrial biomass determined by Mitotracker green calculated using flow cytometry, (n = 6). (b) Mitochondrial membrane potential assessed by Tetramethylrhodamine, ethyl ester (TMRE) using flow cytometry, (n = 6). (c) ATP assessment confirmation using ATP assay kit (Sigma Aldrich, USA), Data is expressed as mean ± SEM (n=3). *P < 0.05, **P < 0.01, ***P < 0.001. (JPG 48 kb) [file 13287_2018_1012_MOESM2_ESM.jpg]
